# Accuracy of a Basketball Indoor Tracking System Based on Standard Bluetooth Low Energy Channels (NBN23^®^)

**DOI:** 10.3390/s18061940

**Published:** 2018-06-14

**Authors:** Bruno Figueira, Bruno Gonçalves, Hugo Folgado, Nerijus Masiulis, Julio Calleja-González, Jaime Sampaio

**Affiliations:** 1 Faculty of Sports Biomedicine, Lithuanian Sports University, 44221 Kaunas, Lithuania; Nerijus.Masiulis@lsu.lt; 2 Research Center in Sports Sciences, Health Sciences and Human Development, CIDESD, 5000-801 Vila Real, Portugal; air.bruno.23@gmail.com (B.G.); hfolgado@uevora.pt (H.F.); ajaime@utad.pt (J.S.); 3 Sport Sciences Department, Universidade de Trás-os-Montes e Alto Douro, 5000-801 Vila Real, Portugal; 4 Departamento de Desporto e Saúde, Escola de Ciências e Tecnologia, Universidade de Évora, 7000 Évora, Portugal; 5 Department of Physical Activity and Sport Science, University of Basque Country, 1007 Vitoria, Alava, Spain; julio.calleja@ehu.es

**Keywords:** position measurement, player tracking, reliability, team sports

## Abstract

The present study aims to identify the accuracy of the NBN23^®^ system, an indoor tracking system based on radio-frequency and standard Bluetooth Low Energy channels. Twelve capture tags were attached to a custom cart with fixed distances of 0.5, 1.0, 1.5, and 1.8 m. The cart was pushed along a predetermined course following the lines of a standard dimensions Basketball court. The course was performed at low speed (<10.0 km/h), medium speed (>10.0 km/h and <20.0 km/h) and high speed (>20.0 km/h). Root mean square error (RMSE) and percentage of variance accounted for (%VAF) were used as accuracy measures. The obtained data showed acceptable accuracy results for both RMSE and %VAF, despite the expected degree of error in position measurement at higher speeds. The RMSE for all the distances and velocities presented an average absolute error of 0.30 ± 0.13 cm with 90.61 ± 8.34 of %VAF, in line with most available systems, and considered acceptable for indoor sports. The processing of data with filter correction seemed to reduce the noise and promote a lower relative error, increasing the %VAF for each measured distance. Research using positional-derived variables in Basketball is still very scarce; thus, this independent test of the NBN23^®^ tracking system provides accuracy details and opens up opportunities to develop new performance indicators that help to optimize training adaptations and performance.

## 1. Introduction

Team sports are very complex to describe, because players perform under a very wide range of environmental information, requiring constant decision making in stressful scenarios [1]. The players’ dynamic behavior under these circumstances is modelled by the ability to identify, interpret, and even predict the actions of teammates and opponents [2]. In this sense, data collection using video and computer tracking systems can be used to generate critical information about movement patterns, allowing in-depth analyses of locomotion [3,4].

Methods of assessing team sport movement data are mostly based on global positioning systems (GPS) [5,6,7], multiple camera methods [8,9] and radio-frequency based systems [10]. In recent years, the usage of radio-frequency based systems is becoming extremely popular, and seems to be providing valuable insights about physical and tactical performance. The measurement of indoor movement displacements, with lower court areas and higher inter-player proximity, seems to affect signal stability in a different way, due to the interference of various information sources around the space [11]. The possible interference with nearby systems that work on the same frequency range, the sensitive procedure of establishing the signal acquisition, the high cost, or the use of independent tags for each player, can be identified as complementary weaknesses of radio-frequency systems [12,13].

Bluetooth-based systems were designed to reduce costs in terms of energy consumption, using small tags (attached to the players) that usually transmit the strength of the received signal to a number of fixed stations, that measure electromagnetic waves to track the final position, using the trilateration technique [14]. Available research has shown the effectiveness of Bluetooth Low Energy channels presenting an average error of 0.5 to 1.0 m [15], due to the low complexity of Trilateration algorithm. For example, the LPM-system is accurate for measuring static and dynamic movements with high validity (correlations ranged between 0.71 and 0.97) despite the progressively increasing error with increases in movement speed [10].

The validation process to assess players’ movement activity is usually accomplished by comparing the calculated individual position data with the real movement, or against a reference system [4,16]. In this field, research is mostly designed to identify the patterns and movement demands of team sports [17]. To the best knowledge of the authors of this paper, no studies have inspected the accuracy of the distance between the tags [18]. In fact, by establishing this accuracy, it would be possible to use positional data with the aim of evaluating tactical performance. Accordingly, this is an emergent topic of research in outdoor team sports such as soccer and rugby, but one that is still unexplored in indoor team sports. The possibilities of research that arise from the use of positional variables allow for the development of new collective performance indicators which are capable of describing the dynamics of the game. For example, processing measures such as the distance to positional-centroid facilitate assessment of inter-player coordination, and depict different predictabilities in players’ movement behavior, that ultimately contribute to overall team organization [19]. However, the literature on Basketball using positional-derived variables is scarce, since player-tracking technology is relatively recent, and is applied exclusively in official NBA games. In this sense, the implementation of an athlete tracking system that could also be used in other competitions and in training sessions may provide fundamental information to optimize training adaptations and performance [20]. Therefore, this study aims to identify the accuracy of the NBN23^®^ system, an indoor tracking system based on standard Bluetooth Low Energy channels (10 Hz).

## 2. Material and Methods

The process of identifying the accuracy of the NBN23^®^ system comprised measuring inter-tag distances using a customized mobile cart. The cart had twelve tags fixed at several known distances (0.5, 1.0, 1.5 and ~1.8 m), and was moved around the basketball court at different speeds (lower than 10.0 km/h; between 10.0 km/h and 20.0 km/h; and higher than 20.0 km/h. Afterwards, the inter-tag distances obtained with the system were compared with the real inter-tag distances.

## 3. Equipment

**Figure 1:**
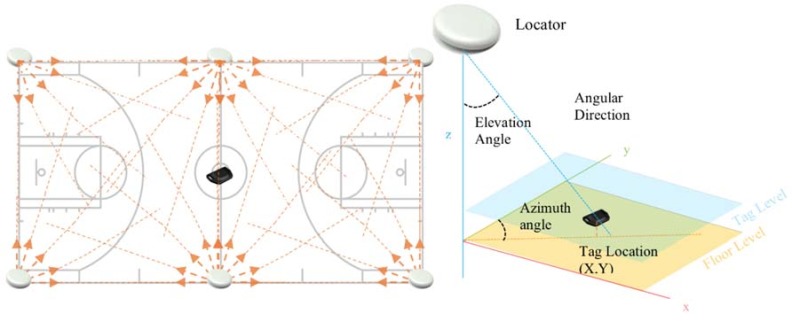
Six advanced Locators (antennas) are used to measure the Angle-of-Arrival transmitted by a Tag.

**Figure 2:**
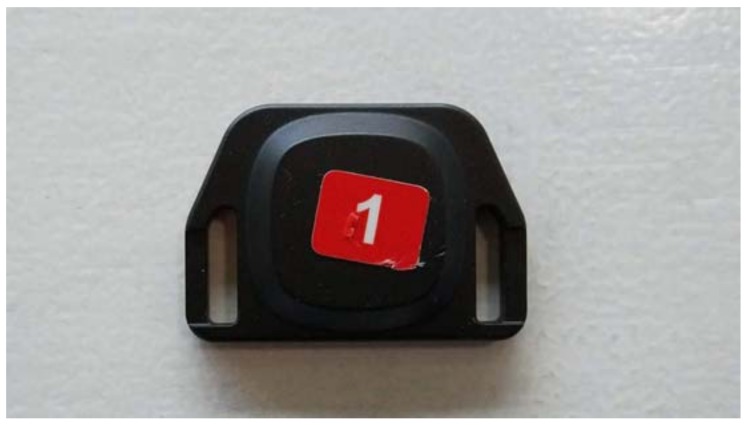
Tags use Bluetooth Low Energy to send a radio signal to a positioning engine, which uses proprietary algorithms to calculate the tag position (44 × 31 × 8 mm, 10 g).

The NBN23^®^ microprocessor system technology is supported by Quuppa Intelligent Locating System™ (RMS accuracy typical 0.5 m; max 0.1 m using a frequency band of 2.4 GHz ISM band, bandwidth of 1 MHz; location event rate of 0.1–10 Hz and a capacity of up to 400 location events per second per channel). This system uses a proprietary technology based on Bluetooth Low Energy (BLE, Bluetooth 4.0 or Bluetooth Smart), and is based on a unique Angle-of-Arrival signal processing method. The Quuppa system uses antennas fixed on the roof of the sports hall (Figure 1), capturing the radio signal emitted by tags carried by the players (Figure 2), and sending it to a positioning engine which uses proprietary algorithms to calculate tag position. The tags have a battery life of 460 h of continuous tracking at 20 Hz, and 9600 h of continuous tracking at 1 Hz, with the following features: triggering with acceleration sensor or optionally with impact switch, locators can adaptively control the transmit rate of individual tags, positioning packets can carry small amount of data (acceleration sensor etc.), and location event rate can be captured at a maximum of 50 Hz. The system is able to provide 2D or 3D positioning, depending on the number of locators (antennas) used. To provide a 2D analysis, one locator is enough; small sports fields can use six (2D) to eight locators (3D), and large sport fields thirty two locators (3D) [21]. The connectivity uses Ethernet and a power of PoE (2 W) or DC 12 V/500 mA. There are two different types of locators target different ranges. The Quuppa system has a collection frequency between 5 and 50 Hz and a latency of around 100 ms [21].

## 4. Data Collection

**Figure 3:**
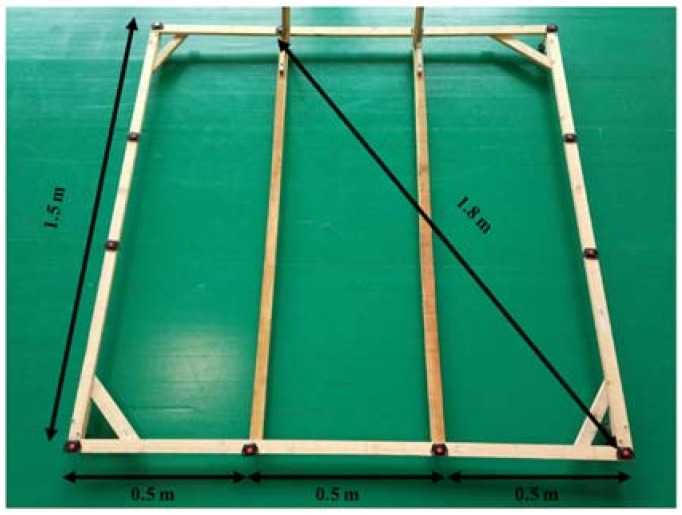
Schematic representation of the custom cart built for tags accommodation and predetermined distances between tags.

**Figure 4:**
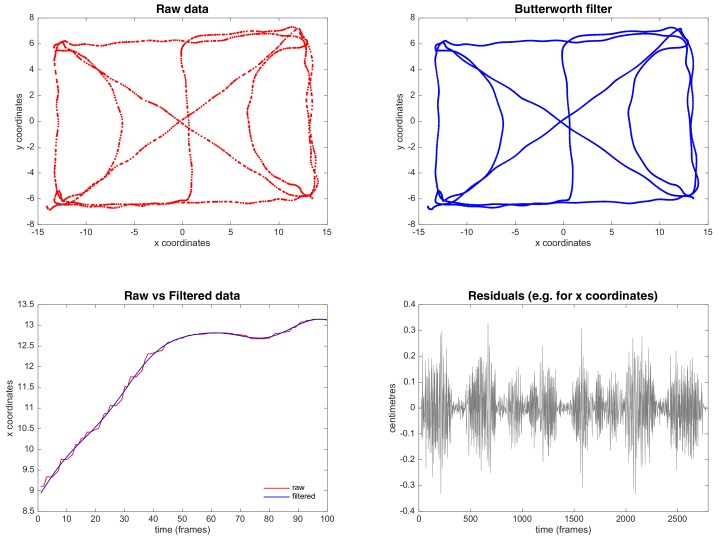
Comparison between raw and smoothing coordinate data, using Butterworth low-pass filter.

The inter-tags’ distance accuracy was calculated using a custom cart with 12 capture tags (Bluetooth Low Energy) attached and placed at ranged distances of 0.5, 1.0, 1.5 and ~1.8 m (1.803 m with 3 decimals) (see Figure 3). The cart was first held motionless during 1 min, and then pushed along a predetermined course in a Basketball court of standard dimensions (the course can be seen in the Figure 4, raw data panel). The course was performed at different speed intensities, and the following ranges were used to analyze the data: low speed (<10.0 km/h), medium speed (>10.0 km/h and <20.0 km/h), and high speed (>20.0 km/h). The cart followed the marked course of the court as closely as possible. After data collection, the positional data (*x* and *y* coordinates) was retrieved from the NBN23^®^ tags and processed in MATLAB^®^ (The Math-Works Inc., Natik, MA, USA). During the data collection, two tags had connection problems and they were excluded from the sample. This decision implied that some data were not considered. Therefore, the final sample comprised eight inter-tags distances for 0.5 m, 6 inter-tags distances for 1.0 m, and 6 inter-tags distances for 1.8 m. Data from each tag were synchronized to ensure the same length of the time series and the positioning dynamic time series were filtered using a fourth-order recursive, zero phase-shift Butterworth low-pass with a cut-off frequency of 3 Hz. Butterworth filters are often chosen for smoothing movement data because they are optimally flat in their pass-band, have relatively high roll-offs, and rapid response in the time domain in the attenuation of the undesired frequencies content. The cut-off frequency was chosen after a series of procedures. First, we performed an analysis of the signal content, in terms of frequencies and their corresponding amplitudes, which showed higher amplitudes in frequencies above 3–5 Hz. Thus, this analysis provided support for the approximate range of cut-off frequencies to be tested in the filtering process. Afterwards, the cut-off frequencies were tested over a range of frequencies lower than 5 Hz, and performed a residual analysis of the filtered and unfiltered signals, as recommended by Winter [22].

## 5. Data Processing and Analysis

The distances between tags were calculated by computing the norm between the vectors, using the following equation:

$$D\left(a_{x_{\left(t\right)},y_{\left(t\right)}},b_{x_{\left(t\right)},y_{\left(t\right)}}\right)=\;\sqrt{{\left(a_{x_{\left(t\right)}}-b_{x_{\left(t\right)}}\right)}^{2}+{\left(a_{y_{\left(t\right)}}-b_{y_{\left(t\right)}}\right)}^{2}}$$

where *D* is the distance between tag1 and tag2, *a* is the tag1, *x* and *y* are the coordinates, *t* is the time, and *b* is the tag2. The inter-tags accuracy was then calculated using the root mean square error (RMSE), using the following equation:

$$\mathrm{RMSE}=\sqrt{\frac{\sum_{t=1}^{n}{\left({\mathrm{Tag}\;\mathrm{distances}}_{t}\;{–\;\mathrm{real}\;\mathrm{distances}}_{t}\right)}^{2}}{n}}$$

where *t* is the time; *n* is the length of considered time series: Finally, the percentage of variance accounted for (%VAF) for each measured distance was calculated using the following equation:

$$\%\;\mathrm{VAF}=100\times \left(1–\frac{\sum_{t=1}^{n}{\left({\mathrm{Tag}\;\mathrm{distances}}_{t}{–\mathrm{real}\;\mathrm{distances}}_{t}\right)}^{2}}{\sum_{t=1}^{n}{\left({\mathrm{Tag}\;\mathrm{distances}}_{t}\right)}^{2}}\right)$$

where *t* is the time; *n* is the length of considered time series. The RMSE was used to quantify the inter-tags linear error, and %VAF was used to quantify how close to the expected values the inter-tags measures were.

## 6. Results

**Figure 5:**
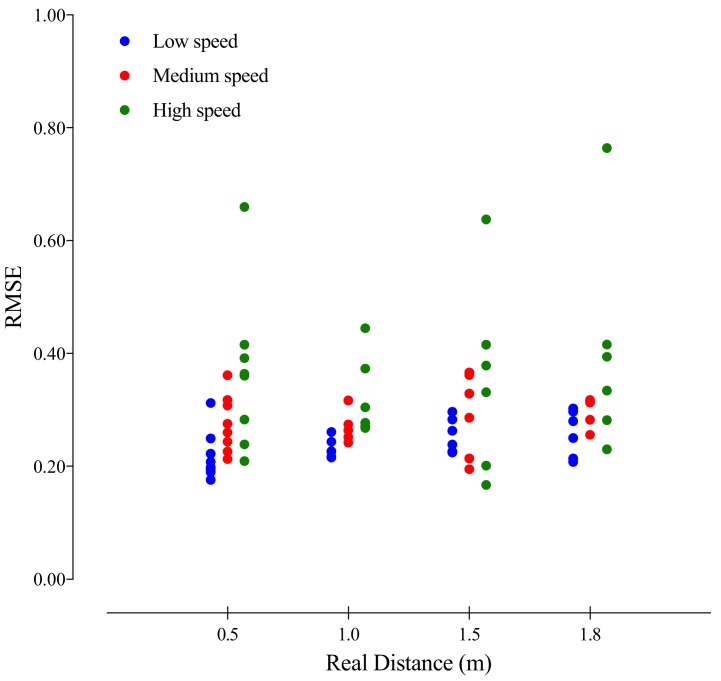
Inter-tag accuracy and the respective predetermined distances between tags.

Within some degree of variation, all distances between tags showed a similar increase in RMSE values when the movement speed increased. Thus, positional data showed higher RMSE at speed movements above 20.0 km/h. Despite that, no particular trend of error alteration was observed; according to different absolute distances between tags, the linear error increases in high speeds, also presenting higher variability between tags (see Table 1 and Figure 5).

The %VAF analysis revealed a tendency for higher accuracy results as absolute distances between tags increased, and an opposite trend when movement speed increased.

The application of the Butterworth low-pass filter showed a smoothing of raw coordinate data, reducing the data noise and improving the accuracy and the quality of interpretation in the movement data reported (see Figure 4).

## 7. Discussion

This study aimed to identify the accuracy of the NBN23^®^ indoor tracking system, using a cart with tags positioned at known distances. Both the accuracy presented by the system manufacturer (Quuppa), of around 0.5 m [21], as well as the reliability results of a similar system (LPM), can be used to contrast the identified accuracy of the NBN23^®^ tracking system. Current results showed that the percentage of variance accounted for (%VAF) tended to dissipate as the distance between tags increased, and that the RMSE is somehow stable regardless of the distance. The mean error presented by the system was 0.30 ± 0.13 m, with the smaller distances between tags (0.5 m) presenting a RMSE of 0.37 ± 0.13 m for high movement speed. The degree of accuracy of the NBN23^®^ indoor tracking system was higher when compared with Bluetooth Low Energy channels results, with average errors between 0.5 and 1.0 m, showing that the present system seems to be sufficiently accurate to track players’ movements [14]. The analysis of %VAF showed lower variance, especially at small distances. In fact, previous research has shown an inadequacy of lower sample units, in particular in collecting high intensity displacements [23]. Overall, the present study showed higher relative accuracy for larger distances. However, the results seem very acceptable for team sports positional analyses, despite the higher dynamic velocity movements presented by players [24].

The design of reliability and validity studies are usually developed using the evaluation of covered distances and speed measurements [25]. However, the inter-unit reliability seems to offer a different perspective in terms of validating the collective information provided by tags. The NBN23^®^ data showed acceptable accuracy results for both RMSE and %VAF when compared with the positioning error indicated by the manufacturer (around 0.5 m), despite the error of the position measurement, that increased with increasing speed.

The obtained results seem to be in line with LPM findings, that presented a mean absolute error of 0.23 ± 0.21 m, and similar increases with accelerations [16]. The analysis of %VAF results showed higher mean relative differences in 0.5 distance between tags, promoting a higher relative accuracy for larger distances. However, for larger distances, our results are similar to the positioning measurements shown by LPM tracking system, which presented an estimated error less than 5% [10].

The acquisition of positional data in sport is often contaminated by various forms of errors that cannot be avoided. However, noise, drift, and outliers can be corrected or mitigated, reducing relative error and improving system accuracy [26]. The LPM system also uses similar techniques to solve position estimation errors, showing more precise estimations [16]. In this sense, the optimization of data using external processing may more reliably reflect the information, and may promote a lower relative error, increasing the %VAF for each measured distance (see Figure 5 and Table 1).

## 8. Conclusions

The results of this study showed that the NBN23^®^ system is acceptable for capturing players’ displacements in indoor team sports such as basketball. Furthermore, the tags are lightweight, shockproof, waterproof, and very easy to attach. The mean error of all NBN23^®^ tracking estimations was 0.30 ± 0.13 m, and the present results are better when compared with average error of 0.5 to 1.0 m presented by the Bluetooth System [15]. Although the mean absolute error increases with increasing movement speeds, the use of external processing filters can correct and mitigate errors, improving system accuracy. Research using positional-derived variables in Basketball is still very scarce; thus, this independent test of the NBN23^®^ tracking system provides accuracy details, and opens up opportunities to develop new performance indicators from an individual and collective basis that help to optimize training adaptations and performance. In addition, the tracking data can also be used to calculate external workload (e.g., distance covered) and provide new interactions with game events, moving towards a more holistic paradigm of collaborative teamwork and game dynamics in basketball.

## Figures and Tables

**Table 1:** Root mean square error and percentage of variance accounted for considered inter-tags distances according to the different movement speed.

Real Distance (meters)	Movement Speed	Root Mean Square Error (meters)	Percentage of Variance Accounted for (%)
0.5 (*n* = 8)	Low (<10 km/h)	0.22 ± 0.04	86.16 ± 3.05
	Medium (10 to 20 km/h)	0.28 ± 0.05	82.27 ± 3.01
	High (>20 km/h)	0.37 ± 0.13	75.86 ± 10.31
1.0 (*n* = 6)	Low (<10 km/h)	0.23 ± 0.02	94.73 ± 0.73
	Medium (10 to 20 km/h)	0.28 ± 0.03	93.22 ± 1.71
	High (>20 km/h)	0.32 ± 0.07	88.82 ± 6.7
1.5 (*n* = 6)	Low (<10 km/h)	0.26 ± 0.03	96.74 ± 0.86
	Medium (10 to 20 km/h)	0.29 ± 0.07	95.9 ± 1.92
	High (>20 km/h)	0.36 ± 0.15	94.15 ± 4.34
1.8 (*n* = 6)	Low (<10 km/h)	0.26 ± 0.04	97.67 ± 0.67
	Medium (10 to 20 km/h)	0.30 ± 0.02	97.19 ± 0.46
	High (>20 km/h)	0.40 ± 0.17	93.76 ± 6.1
